# The early fogging effect in cerebral infarction – a case report and review of the literature

**DOI:** 10.1186/s12883-026-05131-w

**Published:** 2026-07-27

**Authors:** Luisa M. Müller, Daniel Dubinski, Justus Groß, Peter Donndorf, Matthias Wittstock, Daniel Cantré, Thomas M. Freiman, Jens-Christian Schewe, Florian Geßler, Gerd Klinkmann, Sae-Yeon Won

**Affiliations:** 1 Department of Neurosurgery, Rostock University Medical Center, Schillingallee 35, Rostock, 18057 Germany; 2 Department of General and Vascular Surgery, Rostock University Medical Center, Rostock, Germany; 3 Department of Neurology, Rostock University Medical Center, Rostock, Germany; 4 Department of Radiology, Rostock University Medical Center, Rostock, Germany; 5 Department of Anesthesiology, Intensive Care Medicine and Pain Therapy, Rostock University Medical Center, Rostock, Germany; 6 Department of Extracorporeal Therapy Systems, Frauenhofer Institute for Cell Therapy and Immunology IZI, Rostock, Germany

**Keywords:** Fogging effect, Ischemic stroke, Stroke imaging, Neuroimaging artefacts, Cerebellar infarction

## Abstract

**Background:**

The fogging effect is a well-known phenomenon usually seen two to three weeks post stroke, characterized by a pseudonormalization of the infarcted tissue.

**Case presentation:**

A 62-year-old patient underwent emergent thoracic endovascular aortic repair (TEVAR) due to a Stanford Type B aortic dissection. Post-intervention dual antiplatelet therapy and systemic anticoagulation were administered. The following day, the patient developed acute neurological deficits. Cranial CT and perfusion imaging demonstrated an infarction in the left posterior inferior cerebellar artery territory, and conservative management was pursued. A follow-up CT scan 27 h later revealed an isodense area within the infarcted region consistent with the fogging effect.

**Conclusion:**

Our case demonstrated this fogging effect at an unusually early timepoint. We hypothesize that systemic therapeutic anticoagulation together with dual antiplatelet therapy may have contributed to the early onset of fogging. This case highlights the limitations of non-contrast CT in stroke assessment and underscores the importance of multimodal imaging and awareness of this phenomenon.

## Background

Neuroimaging plays a crucial role in the diagnosis and management of ischemic stroke. The choice of imaging modality depends on several factors, particularly the time elapsed since symptom onset. In the clinical setting, the time of symptom onset is often unclear, making appropriate imaging essential to assess salvageable tissue. Computed Tomography (CT) and Magnetic Resonance Imaging (MRI) are the principal imaging modalities used in acute stroke evaluation. Non-contrast CT (NCCT) is a rapid, accessible and useful initial imaging modality, particularly for the exclusion of intracranial hemorrhage, although further imaging may be needed to assess infarction accurately. After approximately 12 to 24 h, NCCT scans typically show hypodensity in the infarcted region. This hypodensity gradually approaches the density of cerebrospinal fluid over time. During this process, typically over several weeks, the infarct may transiently become isodense to surrounding brain parenchyma, obscuring its appearance on NCCT. This phenomenon has been described in the literature as the Fogging Effect [1].

This phenomenon was first described in 1979 by Becker et al. [1], who reported that the infarcted region appeared isodense in NCCT two to three weeks after the event. Approximately 7 days after fogging, the tissue would again appear hypodense [1]. Reports have described variable onset times, ranging from second to third week post-infarction [2, 3]. This artifact, or pseudonormalization, is thought to result from decreased bulk influid, neovascularization, and increased infiltration of erythrocytes and macrophages [1, 2].

Along with the clinical presentation, neuroimaging findings are integral to decision-making in stroke care. Concealment of radiological signs can lead to diagnostic uncertainty and inappropriate management, underscoring the importance of recognizing this phenomenon for radiologists and clinicians. We present a unique case of an unusually early-onset fogging effect.

## Case presentation

The 62-year-old male patient presented to the Emergency Department with acute severe back pain radiating to the thorax and abdomen. At presentation, the patient was hemodynamically stable.

Due to a three-vessel coronary artery disease, previously treated with interventional therapy, the patient was medicated with Acetylsalicylic Acid (ASA). Laboratory testing showed elevated D-dimers of 9070 µl/l, mildly increased inflammation parameters (leucocyte count: 12.43 × 10^9^/l, CRP: 11.0 mg/l) and creatinine (105 µmol/l). Abdominal ultrasound showed no abnormalities. Contrast-enhanced CT revealed an acute Stanford Type B aortic dissection. Neither blood work nor the clinical presentation indicated any organ dysfunction. Based on these findings, the patient was admitted to our Intensive Care Unit (ICU) for blood pressure control and close hemodynamic monitoring. An elective TEVAR procedure was scheduled for the following day.

On the following day, the patient developed acute kidney injury. CT angiography showed distal progression of the dissection involving the right renal artery, with partial renal infarction but preserved perfusion. In addition, imaging demonstrated contrast opacification of the false lumen and collapse of the true lumen, prompting emergent TEVAR. To create an adequate aortic landing zone, the left subclavian artery was occluded using an Amplatzer Vascular Plug (AVP) and covered by the aortic stent graft. The procedure resulted in partial thrombosis of the false lumen with reduced flow.

**Fig. 1:**
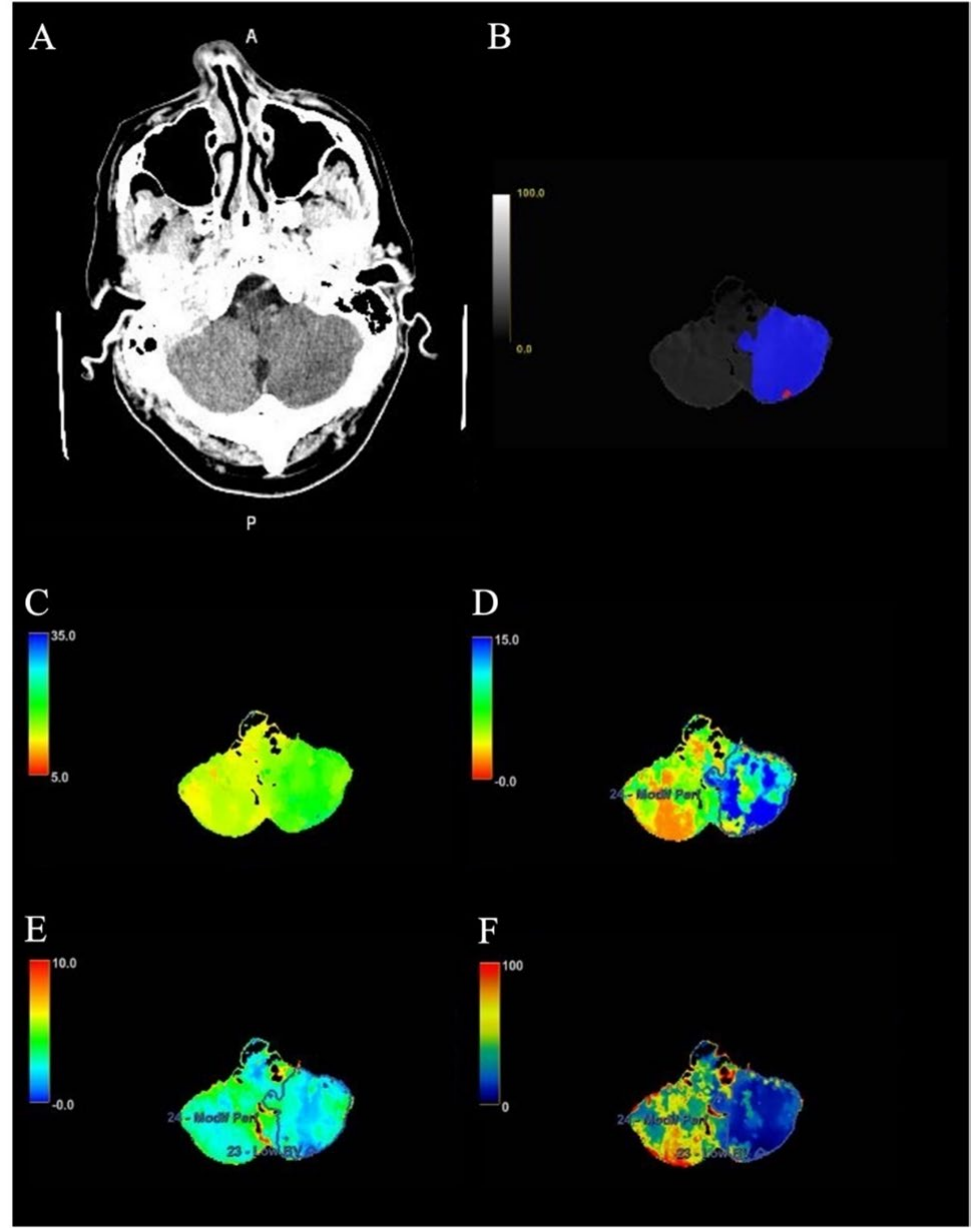
Initial CT and perfusion imaging: Axial NCCT demonstrating a hypodense infarct in the left cerebellar hemisphere **A**; tissue classification map **B**; Time to Peak map demonstrating prolonged perfusion within the infarct territory **C**; Mean Transit Time map showing delayed transit **D**; Cerebral Blood Volume map demonstrating reduced blood volume **E**; Cerebral Blood Flow map demonstrating reduced blood flow within the infarct territory **F**

Following the procedure, the patient was continued on dual antiplatelet therapy, ASA and Clopidogrel, along with systemic anticoagulation targeting a partial thromboplastin time of 40 to 50 s. Within 24 h, the patient developed neurological deficits, including left-sided paralysis, nystagmus, reduced vigilance and impaired spontaneous breathing. Cranial perfusion CT revealed hypoperfusion in the left PICA-territory with a demarcated infarct region (Fig. 1A). In the infarct region, the median transit time (MTT) and time to peak (TTP) were elevated whereas the cerebral blood volume (CBV) and the cerebral blood flow (CBF) were reduced (Fig. 1B-F). The infarct volume measured around 20 ml, and conservative treatment was continued in accordance with recent evidence [4]. Follow-up CT imaging showed no further swelling or evidence of cerebellar herniation, and there was no indication for posterior fossa decompression. Conservative neurocritical care management was continued.

**Fig. 2:**
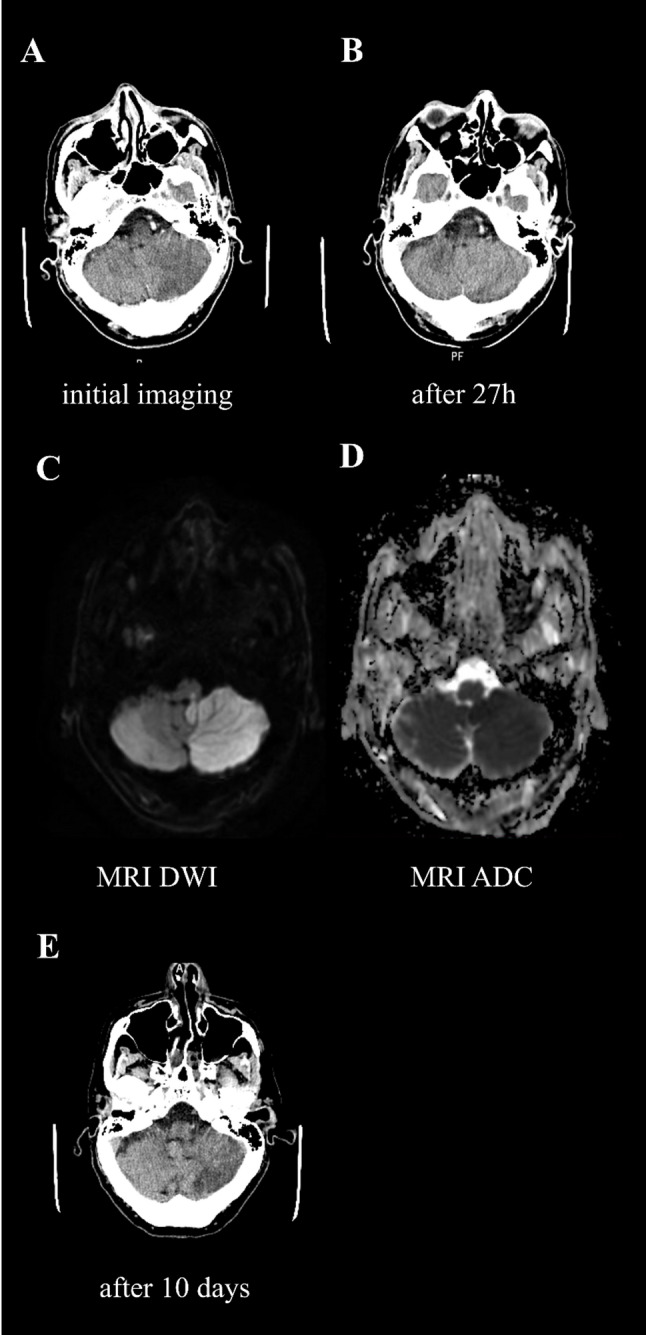
CT and MRI findings during the clinical course: Initial axial NCCT obtained at symptom onset demonstrating a left cerebellar hypodensity **A**; Follow-up axial NCCT performed 27 h later showing disappearance of the hypodensity **B**; MRI DWI (b1000) and corresponding ADC map obtained 57 h later demonstrating a left cerebellar infarction **C **and **D**; axial NCCT performed 10 days after the initial examination showing reappearance of the left cerebellar hypodensity **E**

The CT scan performed 27 h after the initial cranial CT no longer showed the demarcated infarcted area in the left cerebellar hemisphere (Fig. 2B). In absence of improvement of clinical symptoms, this finding raised the suspicion of an early fogging effect. Diffusion-weighted MRI imaging (DWI) on the following day revealed a hyperintense area within the left PICA-territory in the b1000 image (Fig. 2C) with concomitant ADC reduction (Fig. 2D, ADC ~ 520 * 10^− 6^ mm^2^/s vs. ~700 * 10^− 6^ mm^2^/s in the non-affected contralateral cerebellar hemisphere) accompanied by hyperintensity in FLAIR as well as hypointensity in T1-weighted images (not shown), consistent with acute infarction. This supported the interpretation of an early fogging effect. A follow-up CT scan obtained 10 days after the initial imaging demonstrated a well-demarcated infarct in the left PICA-territory (Fig. 2E), further supporting the temporal evolution of the fogging effect. During the subsequent clinical course, the patient developed prolonged respiratory insufficiency requiring tracheostomy. Owing to persistent dysphagia, a percutaneous endoscopic gastrostomy (PEG) tube was also placed. Over the following year, the patient’s condition gradually improved, allowing removal of both the tracheostomy and PEG tube.

### Discussion and conclusion

Since Becker’s initial description [1], multiple reports have documented varying timing of the fogging effect. Pathophysiologically, the fogging phenomenon likely results from a combination of changes in the infarcted tissue [1, 2]: regression of edema, macrophage infiltration, and capillary proliferation, leading to increased density and temporary pseudonormalization on NCCT. Perfusion imaging would show reduced CBV, reduced CBF, and elevated MTT during fogging, similar to chronic infarction. Interestingly, a case series from 2019 investigated three cases in which infarct fogging appeared 6–10 days after symptom onset. All cases showed elevated CBF and CBV, creating the image of a “luxury-like perfusion pattern” [5]. Notably, these infarctions were not caused by thromboembolism but by vasospasms related to subarachnoid or intracerebral hemorrhage. This hyperperfusion would be expected to increase bulk fluid influx rather than decrease it, unlike the mechanism described in previous reports [1, 2].

In our case, the fogging effect occurred unusually early, making the structural and cellular changes typically implicated in infarct fogging less likely. We therefore propose a hypothesis centered on altered perfusion dynamics rather than tissue remodeling. Previous reports have described “luxury perfusion” patterns in association with fogging [5], suggesting that transient hyperperfusion may contribute to pseudonormalization on CT imaging. In the present case, the combination of dual antiplatelet therapy and systemic anticoagulation, together with altered vertebrobasilar hemodynamics following TEVAR and subclavian artery occlusion, may have influenced microvascular perfusion. Unfortunately, perfusion imaging was not available at the time the fogging effect was observed. Nonetheless, it is conceivable that systemic anticoagulation may have influenced hyperperfusion via recanalization or microvascular changes, similar to previously described cases [5]. However, this interpretation remains speculative and should be considered hypothesis-generating. Early fogging is not commonly reported in routine thrombolysis or anticoagulation, suggesting that additional factors – such as infarct location, collateral circulation, or specific hemodynamic conditions – may contribute.

A previous study published in 2017 by Dekeyzer et al. [6] evaluated infarct fogging on imaging immediately after endovascular stroke treatment (EST). In this study, contrast leakage was discussed as a possible explanation. CT scans of patients who showed cerebral infarction as a hypodensity were followed up over a short period of time. All patients in this study received endovascular treatment and in 24.3% of cases, the stroke area presented isodense in postinterventional CT scans. In the short-term repeat CT scan 4.5 h after the procedure, the infarct demarcation reappeared in all patients again. This very short-term phenomenon was attributed to contrast leakage after endovascular intervention which is similar to the commonly observed postinterventional hyperdensities [7]. Another important differential diagnosis is early spontaneous recanalization of the affected vascular territory, which may lead to normalization of CT attenuation and mimic the fogging effect. In contrast, no endovascular treatment was performed at the time of fogging and persistent lesion on MRI with reappearance of hypodensity on follow-up CT after 10 days was observed in our case supporting the interpretation of fogging rather than sustained reperfusion or tissue recovery.

Because the decision to perform surgical intervention is often based on clinical deterioration, a surgical approach might have been chosen in this case. A recent publication [4] showed that the superior treatment is based on the infarct volume. Thus, a conservative treatment was chosen, as the infarcted volume measured only 20 ml. Cerebellar infarctions may exhibit the fogging effect more frequently than recognized, but early surgical intervention in many centers may obscure its detection.

NCCT is often used in stroke follow-up to evaluate infarction size and guide secondary prevention, including anticoagulation decisions. Early fogging may lead to underestimation of infarct size and impair decision-making, highlighting its diagnostic relevance. Prognostic relevance, however, remains unknown and requires further research, especially in larger samples. The time variation likely depends on several factors, such as tissue remodeling, anticoagulation, other medications, blood-brain barrier integrity, and lesion location. If early fogging occurs more frequently than currently recognized, reliance on NCCT alone may lead to underestimation of infarct extent. Further diagnostic testing may therefore be necessary to devise an appropriate treatment plan, which is why further understanding of this phenomenon is needed.

The fogging effect represents a diagnostic challenge after ischemic stroke, especially when no previous imaging for comparison is available. Awareness of the phenomenon is crucial to avoid misdiagnosis and subsequent inappropriate clinical decision-making. Underestimation of infarct size can influence therapeutic strategies, such as timing of anticoagulation or neurosurgical intervention. NCCT scans alone may be insufficient to reliably assess infarct extent, particularly in patients undergoing therapeutic anticoagulation; therefore, multimodal imaging is recommended. The factors influencing the occurrence and timing of the fogging effect remain unclear. Prospective studies using a high frequency of multimodal imaging are necessary to clarify the frequency, timing, pathophysiology, and correlation with clinical outcome. A deeper understanding of the fogging effect could enhance diagnostic accuracy and improve management strategies in acute and subacute ischemic stroke.

## Data Availability

Upon reasonable request, the data can be provided.

## **Abbreviations**

ADC: Apparent Diffusion Coefficient
ASA: Acetylsalicylic Acid
AVP: Amplatzer Vascular Plug
CBF: Cerebral Blood Flow
CBV: Cerebral Blood Volume
CRP: C-reactive Protein
CSF: Cerebrospinal Fluid
CT: Computed Tomography
DWI: Diffusion weighted images
EST: Endovascular stroke treatment
ICU: Intensive Care Unit
MRI: Magnetic Resonance Imaging
MTT: Median Transit Time
NCCT: Non-Contrast Computed Tomography
PEG: Percutaneous endoscopic gastrostomy
PICA: Posterior Inferior Cerebellar Artery
TEVAR: Thoracic Endovascular Aortic Repair
TTP: Time To Peak
